# Evaluation of methods to eliminate analytical interference in multiple myeloma patients with spurious hyperphosphatemia

**DOI:** 10.1093/labmed/lmad012

**Published:** 2023-03-21

**Authors:** Xin Tian, Li Zhao, Jin Ma, Jie Lu, Tian-yi Zhu, Yan Liu, Hong-xun Sun

**Affiliations:** Department of Laboratory Medicine, The Third Hospital of Hebei Medical University, Shijiazhuang, China; Department of Laboratory Medicine, The Third Hospital of Hebei Medical University, Shijiazhuang, China; Department of Laboratory Medicine, The Third Hospital of Hebei Medical University, Shijiazhuang, China; Department of Laboratory Medicine, The Third Hospital of Hebei Medical University, Shijiazhuang, China; Department of Laboratory Medicine, The Third Hospital of Hebei Medical University, Shijiazhuang, China; Department of Laboratory Medicine, The Third Hospital of Hebei Medical University, Shijiazhuang, China; Department of Laboratory Medicine, The Third Hospital of Hebei Medical University, Shijiazhuang, China

**Keywords:** paraprotein interference, spurious hypophosphate, multiple myeloma

## Abstract

**Objective:**

The acid/molybdate assay performed on the Beckman Coulter AU5821 could be subject to paraprotein interference, which potentially results in spurious hyperphosphatemia. We attempted to find a reliable solution to eliminate paraprotein interference in laboratory test results and discuss the causes of paraprotein interference.

**Methods:**

We observed 50 multiple myeloma patients with serum paraproteins. We used the trichloroacetic acid (TCA) deproteinizing method to confirm that paraproteins indeed interfered with phosphate detection in the serum acid/molybdate assay. Furthermore, we used the dry chemical method (Vitros 5.1 FS, Johnson) and deionized water (H_2_O), normal saline (NS), and healthy human serum as alternative diluents. We assessed the clinical acceptability of the 4 methods by evaluating a bias percentage (bias%) lower than 10% under the premise of TCA treatment as a serum phosphate reference method.

**Results:**

In total, comparing the results of the TCA treatment on the Beckman Coulter AU5821, 3/50 (6%) multiple myeloma patients exhibited phosphate pseudo-elevation (bias% >10%). Additionally, we found pseudo-hypophosphate only in immunoglobulin (Ig)G-kappa paraprotein samples, and all were above 50 g/L. The bias% between TCA and dry chemical method for the 3 patients was below 10%. The maximum acceptable dilutions for patient 22 were 8-fold H_2_O, 4-fold H_2_O , and 2-fold serum; those for patient 45 were 16-fold H_2_O, 16-fold H_2_O, and 2-fold serum. However, the bias% of patient 40 was beyond the acceptable range in all 3 dilution groups.

**Conclusion:**

High concentrations of IgG kappa–type paraproteins are more likely to interfere with serum phosphorous detection. Both the TCA and dry chemical method can effectively eliminate paraprotein interference.

Paraproteins are monoclonal immunoglobulins (Igs) or immunoglobulin fractions present in the blood or urine produced by a clonal population of B-cell lineage cells, most commonly plasma cells. The presence of paraproteins may signify a variety of underlying conditions, ranging from a benign process known as monoclonal gammopathy of unknown significance to plasma cell malignancy, that is, multiple myeloma (MM).^1^ It has been reported that paraproteins often interfere with biochemical immunoassays and result in inaccurate test results.^2,3^ In particular, there have been several reports indicating that serum phosphate concentrations may be erroneously high in patients with paraproteinemia if the method of phosphate measurement is based on a direct reaction with molybdate in an acid medium.^4^ However, the method information sheet accompanying the current phosphate method kit does not explicitly mention the possibility of interference by paraproteins. Therefore, the presence of pseudo-hyperphosphorus interference by paraprotein effects could be more widespread than realized due to current laboratory testing method limitations.

Hyperphosphatemia occurs in hypoparathyroidism, acute rhabdomyolysis, and metabolic acidosis. Hyperphosphatemia also occurs in secondary renal damage caused by B-cell neoplasia, such as MM or Waldenström’s macroglobulinemia.^5^ If incorrect serum phosphate results are reported to clinicians, they could lead to unnecessary investigations and changes to patient treatment. Therefore, in the current study, serum phosphate concentrations were measured in 50 MM patients with serum paraproteins to study how paraproteins interfered with phosphate detection in the serum direct acid/molybdate method. We attempted to find a reliable and simple solution to eliminate paraprotein interference for laboratory serum phosphate examination and further explore the causes of paraprotein interference by using the trichloroacetic acid (TCA) deproteinizing method to remove paraproteins to accurately assay the serum phosphate and used deionized water (H_2_O), normal saline (NS), and healthy low-value human serum as alternative diluents.

## Materials and Methods

### Ethics Statement

All procedures performed in the study involving human participants were in accordance with the ethical standards of the institutional and/or national research committee and with the 1964 Helsinki Declaration and its later amendments or comparable ethical standards. The study protocol was approved by Hebei Medical University ethical committee (W2021-095-1). Written informed consent was obtained from each participant.

### Study Subjects

Subjects meeting the following criteria were enrolled in the study: (1) Chinese patients who met the diagnosis standard for MM according to the Revised International Myeloma Working Group criteria (IMWG 2014)^6^; and (2) MM patients with a monoclonal immunoglobulin band identified by serum protein electrophoresis. Patients whose serum samples were lipemic, icteric, or hemolytic or those who were treated with phosphate therapy were excluded.

### Paraprotein Typing and Concentration Detection

Identified paraproteins were typed by immunofixation electrophoresis as IgM, IgG, IgE, IgA, or IgD, and light chain type (kappa or lambda). The paraprotein concentration was detected by the immunoturbidimetric method on the Beckman Coulter IMMAGE 800 Specific Protein Analyzer.

### Methods for Serum Phosphate Concentration Detection

#### Beckman Coulter AU5821 Determination Method

Serum phosphate concentrations were analyzed using a Beckman Coulter AU5821 and an ammonium molybdate–based method in which inorganic phosphorus reacted with ammonium molybdate in the presence of sulfuric acid to form an unreduced phosphomolybdate complex, which was measured as an end-point reaction at 340 nm (reference interval: 0.85–1.51 mmol/L).

#### TCA Deproteinizing Method

Samples were deproteinized using TCA (Deproteinizing Sample Preparation Kit, Bio Vision).The protocol included: (1) Protein precipitation, in which 150 μL sample was mixed with 15 μL cold TCA in a 1.5 mL microcentrifuge tube. The sample was kept on ice for 15 min, then centrifuged at 12,000g for 5 min. Supernatant was carefully transferred to another tube. (2) Sample neutralization, in which 10 μL cold neutralization solution was added to the collected supernatant. The sample was placed on ice for 5 min and directly measured with the Beckman Coulter AU5821 analyzer for the phosphate concentrations. We defined the serum phosphate results by the TCA deproteinizing method and removal of paraproteins as the true result and compared them with the original results to confirm whether there were pseudo-hyperphosphatemia test results in patients with MM with paraproteins, where the relative bias (bias%) between the 2 assays was more than 10%. We analyzed the relationship between paraprotein-interfered serum phosphate determination samples and paraprotein concentrations and immunoglobulin typing.

### Methods for Removing Paraprotein Interference

#### Vitros 5.1 FS Detection Method

Serum phosphate concentrations were analyzed using the dry chemical method (Vitros 5.1 FS, Johnson). Vitros 5.1 FS analysis requires the use of a slide-based method for the reaction of inorganic phosphorus with ammonium molybdate to form a phosphomolybdate complex. This complex was then reduced by p-methyl-aminophenol sulfate, an organic reductant, to form a stable heteropoly molybdenum blue chromophore. The phosphate concentration was then estimated by reflectance spectrophotometry at 670 nm.

#### Dilution Methods

Diluents included H_2_O, NS, and healthy low-value serum phosphorus samples; the original serum samples containing paraproteins were diluted by the various diluents, and serum phosphate was remeasured on the AU5821 analyzer.

Separately, we compared the results by the multiple methods for removing paraprotein interference with the results by TCA treatment, where a bias% <10% was the judgment criterion, to determine the most desirable method to detect serum phosphorus interference by paraproteins in daily practice.

### Statistical Methods

Continuous variables conforming to a normal distribution are described as mean ± SD; otherwise, they are described as median and interquartile range (P_25_–P_75_). Categorical variables are described as frequencies and percentages (%).

The absolute bias was defined by the difference between the serum phosphate results by the TCA deproteinizing method and the original serum phosphate concentrations by AU5821. The relative bias (bias%) denoted the absolute bias divided by the original serum phosphate concentrations by AU5821. If the bias% was more than 10%, there was a difference; that is, there was pseudo-hyperphosphorus interference by paraprotein effects in the AU5821 analyzer.

## Results

### Patient Characteristics and Paraprotein Typing

In the current study, 50 different MM patients were included, 23 females and 27 males. The median age was 63 years (interquartile range, 43-80 years). Of the study patients, 27 had IgG typing paraproteins (9 IgG lambda and 18 IgG kappa), 18 had IgA paraproteins (10 IgA lambda and 8 IgA kappa), and 5 had IgM paraproteins (4 IgM kappa and 1 IgM lambda). The paraprotein concentration range was 5.62 to 74.90 g/L with a median of 23.9 (12.06–40.31) g/L  (**Table 1**).

**Table 1.** Basic Characteristics of 50 Patients with Multiple Myeloma

Patient No.	AU5821	TCA	Bias%	Paraprotein type	Paraprotein concentration (g/L)	Patient No.	AU5821	TCA	Bias%	Paraprotein type	Paraprotein concentration (g/L)
1	1.41	1.38	2%	IgG kappa	18.64	26	0.91	0.93	-2%	IgM kappa	8.26
2	1.50	1.46	3%	IgG lambda	8.53	27	1.34	1.39	-4%	IgG lambda	11.82
3	1.29	1.21	6%	IgA kappa	5.62	28	0.92	0.91	1%	IgG lambda	31.27
4	2.28	2.18	5%	IgM lambda	7.15	29	1.27	1.30	-2%	IgG lambda	17.16
5	1.34	1.45	-8%	IgG kappa	43.85	30	0.90	0.93	-3%	IgA lambda	34.20
6	0.99	0.92	7%	IgA lambda	31.90	31	1.18	1.20	-2%	IgG kappa	23.70
7	1.33	1.24	7%	IgG kappa	31.3	32	1.18	1.23	-4%	IgA lambda	55.70
8	1.48	1.34	10%	IgG kappa	38.39	33	1.32	1.26	4%	IgG kappa	25.47
9	1.31	1.28	3%	IgG kappa	13.54	34	0.92	0.99	-7%	IgA kappa	23.90
10	1.42	1.33	7%	IgA lambda	38.91	35	1.26	1.26	0%	IgA kappa	25.00
11	1.04	1.00	4%	IgA lambda	15.3	36	0.96	1.00	-4%	IgG kappa	8.70
12	1.79	1.61	10%	IgA lambda	57.4	37	1.00	1.00	0%	IgG kappa	15.40
13	1.34	1.25	7%	IgA kappa	60.42	38	0.69	0.63	9%	IgM kappa	7.5
14	1.05	1.13	-7%	IgG lambda	16.73	39	0.93	0.96	-3%	IgG kappa	8.9
15	0.83	0.88	-5%	IgA kappa	10.77	**40**	**5.50**	**2.25**	**59%**	**IgG kappa**	**73.90**
16	1.63	1.61	1%	IgA lambda	13.78	41	1.27	1.38	-8%	IgA kappa	60.3
17	1.34	1.35	-1%	IgA lambda	12.62	42	1.61	1.54	5%	IgA lambda	37.1
18	1.35	1.43	-6%	IgG kappa	74.90	43	0.74	0.75	-1%	IgG lambda	19.09
19	1.30	1.23	6%	IgA lambda	26.28	44	1.49	1.44	4%	IgG kappa	53.9
20	1.50	1.48	2%	IgM kappa	38.12	**45**	**3.19**	**1.56**	**51%**	**IgG kappa**	**50.10**
21	1.83	1.86	-2%	IgG lambda	34.20	46	1.41	1.46	-4%	IgG kappa	6.97
**22**	**2.96**	**1.56**	**47%**	**IgG kappa**	**59.90**	47	1.45	1.46	-1%	IgA kappa	12.3
23	2.50	2.26	10%	IgG kappa	46.65	48	1.46	1.36	7%	IgG kappa	41.7
24	1.42	1.43	0%	IgM kappa	8.07	49	1.53	1.41	8%	IgA kappa	9.5
25	1.35	1.26	6%	IgG lambda	22.79	50	1.47	1.46	1%	IgG lambda	49.3

AU5821, the serum phosphate concentration(mmol/L) result measured on the AU5821 analyzer; TCA, the serum phosphate concentration(mmol/L) result measured on AU5821 after TCA precipitation method.

### Original Serum Phosphate Results and TCA Treatment Results

Of the 50 paraprotein-positive samples, 3 (6%) showed a bias% greater than 10% and phosphate pseudo-elevation. In addition, we found pseudo-hyperphosphate only in IgG kappa–type paraprotein samples. The concentrations of paraproteins in the 3 cases were all above 50 g/L, and serum total protein was above 100 g/L (**Table 1** and **Table 2**).

**Table 2.** Patient Characteristics Associated with Spurious Hyperphosphatemia and Results After Dilution

Characteristic	22	40	45
	Patient No.		
Sex	M	M	M
Age (y)	57	62	66
Monoclonal component type	IgG kappa	IgG kappa	IgG kappa
Monoclonal component (g/L)	59.90	73.90	50.10
Serum total protein (g/L)	118.09	121.29	118.56
Serum creatinine (mmol/L)	57.16	544.79	99.92
Serum calcium (mmol/L)	2.18	2.88	2.17
Serum phosphorus by AU5821 (mmol/L)	2.96	5.50	3.19
Serum phosphorus by TCA (mmol/L)	1.56	2.25	1.75
Serum phosphorus by Vitros (mmol/L)	1.69	2.43	1.81

IgG, immunoglobulin.

### Comparison of the Results of the Dilution Group and Vitros Group with TCA

The maximum dilutions of patient 22 were 8-fold H_2_O, 4-fold NS, and 2-fold low-value phosphate serum; those for patient 45 were 16-fold H_2_O, 16-fold NS, and 2-fold serum. However, the bias% of patient 40 was beyond the acceptable range in the 3 dilution groups. The bias% of the Vitros 5.1 FS method compared with the TCA method was less than 10% in these 3 cases (**Table 3**).

**Table 3.** Recovery Rate of Serum Phosphate on the AU 5821 Analyzer at Various Dilutions

		H_2_0			NS			Serum		
	Dilution	Expected value (mmol/L)	Obtained value (mmol/L)	Bias (%)	Expected value (mmol/L)	Obtained value (mmol/L)	Bias (%)	Expected value (mmol/L)	Obtained value (mmol/L)	Bias (%)
Patient 22	Original	1.56	—	—	1.56	—	—	1.56	—	—
	2×	0.78	0.78	0.00^a^	0.78	0.85	8.97^a^	0.78	0.82	5.13^a^
	4×	0.39	0.37	– 5.13^a^	0.39	0.36	– 7.69^a^	0.39	0.34	–12.82
	8×	0.20	0.18	– 7.69^a^	0.20	0.16	– 17.95	0.20	0.16	–17.95
	16×	0.10	0.08	– 17.95	0.10	0.08	– 17.95	0.10	0.07	–28.21
Patient 40	Original	2.25	—	—	2.25	—	—	2.25	—	—
	2×	1.13	3.16	180.89	1.13	3.34	196.89	1.13	3.97	252.89
	4×	0.56	2.20	291.11	0.56	1.19	111.56	0.56	1.16	105.99
	8×	0.28	0.76	170.22	0.28	0.74	163.11	0.28	0.66	133.29
	16×	0.14	0.18	28.00	0.14	0.18	28.00	0.14	0.16	14.13
Patient 45	Original	1.75	—	—	1.75	—	—	1.75	—	—
	2×	0.88	0.96	9.71^a^	0.88	0.95	8.57^a^	0.88	0.94	7.43^a^
	4×	0.44	0.46	5.14^a^	0.44	0.43	– 1.71^a^	0.44	0.39	–10.86
	8×	0.22	0.20	– 8.57^a^	0.22	0.20	– 8.57^a^	0.22	0.18	–17.71
	16×	0.11	0.10	– 8.57^a^	0.11	0.10	– 8.57^a^	0.11	0.09	–17.71

^a^Indicates that the bias% is within the acceptable range.

## Discussion

Hyperphosphatemia is usually secondary to hypoparathyroidism or advanced renal failure. Pseudo-hyperphosphatemia, a rare condition, is most commonly associated with paraproteinemia but is also seen in other conditions, such as hyperlipidemia, hyperbilirubinemia, and hemolysis. Pseudo-hyperphosphatemia is found in MM samples, caused by the interaction of the paraprotein with molybdate reagent in the acid molybdate-based assay.^7–11^ Indeed, in the current study, we discovered 3 cases of pseudo-hyperphosphatemia in our laboratory from April 2021 to January 2022, all of them IgG kappa–type MM. However, the available studies do not suggest that the pseudo-increased serum phosphorus has a significant correlation with paraprotein types. Sinclair et al^12^ reported IgA and IgG with pseudo-increased blood phosphorus in MM patients. In 2007, Kiki et al^9^ reported another pseudo-elevation in serum phosphorus in IgG MM. In 2015, Made et al^13^ found pseudo-hyperphosphatemia in an IgA kappa MM patient. Additionally, the study results are discrepant about whether the paraprotein concentration is related to the spurious increase in phosphorus. In the current study, we found that the paraprotein concentration of 3 cases was more than 50 g/L, indicating that high concentrations of paraprotein are more likely to cause interference. Some experts also believe that paraprotein interference is more likely to occur at high paraprotein concentrations.^14^ However, Roy^1^ insisted that there is no significant correlation between paraprotein interference and concentration or type.

Furthermore, it has been reported that artifactual laboratory abnormalities are uncommon; percentage of abnormalities were found to be 1.2% and 1.5% in 2 studies.^10,11^ We found that of the 50 paraprotein-positive samples, 3 (6%) exhibited phosphate pseudo-elevation. The incidence of such a phenomenon is not accurately known and may be even higher than generally appreciated. It is important for clinicians to be aware of the possibility and recognize artifactual errors in laboratory parameters so that unnecessary tests and erroneous conclusions can be avoided. The wide variation in reported incidences of pseudo-hyperphosphatemia may be due to different definitions of the phenomenon in different studies. We defined any phosphate value measured on the AU5821 that was 10% different from the result obtained on TCA as being pseudo-hyperphosphatemia. Because TCA could completely eliminate the paraproteins, we treated the TCA result as the real result. There was no significant difference in the serum phosphate values between the TCA treatment and the Vitros 5.1 FS dry chemical analyzer method, indicating that both methods had a similar ability to resist paraprotein interference in the clinical laboratory. The Vitros 5.1 FS method is reported to be interference-free because the sample has to penetrate several slide layers before reaching the reaction layer, although the method is based on the reaction of phosphate with ammonium molybdate. This may therefore have the effect of filtering out any interfering compounds.^15^ Finally, we attempted the dilution method commonly used in eliminating analytical interference and compared it with the TCA method. The maximum acceptable dilutions of patient 22 were 8-fold H_2_O, 4-fold NS, and 2-fold low-value phosphate serum and those of patient 45 were 16-fold H_2_O, 16-fold NS, and 2-fold serum. The only exception was patient 40, whose results lacked linearity. This patient’s phosphate concentrations measured on the AU5821 system were 5.5 mmol/L and the overestimated values were absurdly high. In addition, the patient had severe renal impairment (creatinine: 544.79 mmol/L). As a result, this sample is not linear with the TCA method regardless of dilution method. However, the TCA treatment result of this sample was 2.25 mmol/L, and the dry chemical result was 2.43 mmol/L. These results are a more realistic reflection of the patient’s physiological state.

The purpose of the current study was to analyze the clinical laboratory abnormalities caused by paraproteins to improve awareness of the possibility of such interference and avoid unnecessary erroneous conclusions in practical work. When suspicious results appear, the TCA method is preferred for processing samples. If a laboratory does not have a deproteinizing reagent, the dry chemical (Vitros) method is reliable. Through a series of dilutions, the results were also close to the real results except for an unexpectedly high concentration of phosphorus. It is very important to provide clinicians and patients with more accurate results, which can assist clinicians in making accurate diagnoses.

## Conclusion

A high concentration of paraproteins is more likely to cause interference, and IgG kappa–type paraproteins are more likely to interfere with phosphorous detection. Both the TCA and dry chemical methods can effectively eliminate paraprotein interference. Paraprotein interference cannot be completely eliminated by dilution, especially with very high paraprotein concentrations.

## Data Availability

The datasets used and/or analyzed during the current study are available from the corresponding author on reasonable request.

## Abbreviations

TCA: trichloroacetic acid
H_2_O: deionized water
NS: normal saline
bias%: bias percentage
Ig: immunoglobulin
MM: multiple myeloma
